# Slow Stretching That Mimics Embryonic Growth Rate Stimulates Structural and Mechanical Development of Tendon-Like Tissue In Vitro

**DOI:** 10.1002/dvdy.22760

**Published:** 2011-10-18

**Authors:** Nicholas S Kalson, David F Holmes, Andreas Herchenhan, Yinhui Lu, Toby Starborg, Karl E Kadler

**Affiliations:** Wellcome Trust Centre for Cell-Matrix Research, Faculty of Life Sciences, University of ManchesterManchester, United Kingdom

**Keywords:** bio-artificial tendon, tendon, collagen, fibril, development, mechanical properties, electron microscopy, 3D-reconstruction, tension, elasticity

## Abstract

A distinctive feature of embryonic tendon development is the steady increase in collagen fibril diameter and associated improvement of tissue mechanical properties. A potential mechanical stimulus for these changes is slow stretching of the tendon during limb growth. Testing this hypothesis in vivo is complicated by the presence of other developmental processes including muscle development and innervation. Here we used a cell culture tendon-like construct to determine if slow stretch can explain the increases in fibril diameter and mechanical properties that are observed in vivo. Non-stretched constructs had an ultrastructural appearance and mechanical properties similar to those of early embryonic tendon. However, slowly stretching during 4 days in culture increased collagen fibril diameter, fibril packing volume, and mechanical stiffness, and thereby mimicked embryonic development. 3D EM showed cells with improved longitudinal alignment and elongated nuclei, which raises the hypothesis that nuclear deformation could be a novel mechanism during tendon development. Developmental Dynamics 240:2520–2528, 2011. © 2011 Wiley Periodicals, Inc.

## INTRODUCTION

Tendons are fibrous connective tissues that provide a mechanical attachment of muscles to bone. The formation of a mechanically functional tendon requires progressive structural changes to the extracellular matrix (ECM) throughout embryonic and post-natal development. The result is an ECM-rich tissue containing large-diameter collagen fibrils that can withstand forces exerted during everyday locomotor activities. The formation of the large-diameter fibrils occurs in two distinct phases. Phase 1 occurs during embryonic development and involves a gradual and uniform increase in diameters from ∼30 to ∼40 nm and an increase in fibril volume fraction (i.e., the fraction of the tissue occupied by collagen fibrils). Phase 2 occurs postnatally and involves a change from the unimodal distribution of fibril diameters characteristic of embryonic development to a bimodal diameter distribution (Humphries et al.,^2008^). Phase 2 occurs soon after birth in the absence of appreciable limb lengthening. However, Phase 1 is slow and progresses throughout embryonic tendinogenesis (∼6 days in chick and mouse) when the limbs of the animals double in length in a few days. The mechanical changes that these structural changes cause are an increase in the modulus of elasticity and ultimate tensile strength by two orders of magnitude (McBride et al.,^1985^,^1988^). The mechanisms underlying the structural and mechanical changes during Phase 1 remain poorly understood. This is because of the challenges of studying collagen fibril assembly and mechanical property changes in the tendon in vivo, set against a background of gross anatomical changes including vascularization and innervation of the tendon as well as tissue interactions involving the tendon synovium, muscle, and bone.

We have developed a culture system in which cells from embryonic chick metatarsal tendon synthesize a tendon-like construct when cultured in fixed-length fibrin gels (Kapacee et al.,^2008^). Investigations using electron microscopy and material testing revealed that the tendon-like constructs have the structural appearance and mechanical behaviour of native embryonic tendon (Kalson et al.,^2010^). Furthermore, tendon-like constructs increase in mechanical strength and stiffness during 7–10 days post-formation but the improvement does not continue with longer culture times. The limited improvement in mechanical properties of the tendon-like constructs with time in culture, as well as the absence of fibril diameter increases, raised the possibility that externally-driven progressive strain (which was absent in the original design of the culture environment) could be a primary stimulus to invoke the structural and mechanical changes that are seen in vivo. The developing chick leg grows rapidly during gestation, approximately doubling in length between embryonic day 10 and 14 (Hamburger and Hamilton,^1992^). We hypothesised that this growth is a stimulus for the structural and material changes seen in developing tendon and built a new rig to replicate the strain rate in culture.

Mechanical loading of tendon during development occurs both from muscular activity and from growth-related elongation of bones. The strain rates associated with these two types of load differ by approximately four orders of magnitude and are expected to elicit different cellular responses. The effect of muscle-induced loading has been extensively studied by applying cyclic loading to isolated tissues and cell culture systems (Waggett et al.,^2006^; Chokalingam et al.,^2009^; reviewed in Wang and Thampatty,^2008^; Wozniak and Chen,^2009^). In contrast, little is known about the effects of steady strain rates on collagen fibril diameter and mechanical properties of a tendon-like tissue. Here we report that the application of steady strain to tendon-like constructs results in changes in the collagenous matrix that resemble those that occur during embryonic development with associated improvement in mechanical properties.

## RESULTS

**Fig. 1:**
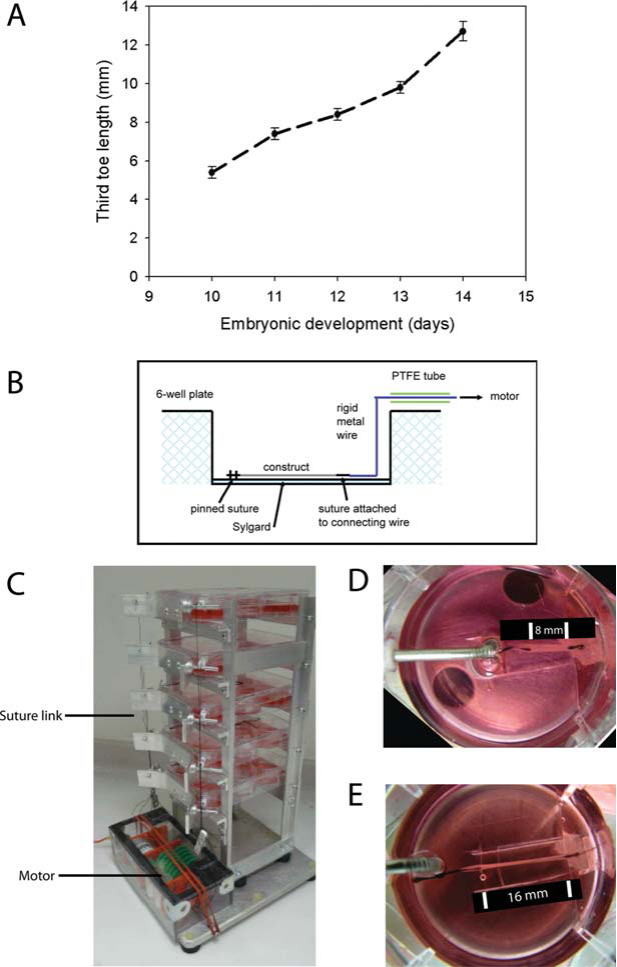
The stretching rig. **A:** Growth of the chick third toe during development. Between embryonic day 10 and day 14, the third toe increases in length from 5.9±0.3 mm to 12.9±0.4 mm. Data reproduced from (Hamburger and Hamilton,^1992^). **B:** Schematic diagram of the well plate setup. Tendon-constructs were connected directly to the low-geared motor by a taut suture link onto a slowly rotating axle. **C:** The stretching rig. Constructs to be stretched were linked to a slowly turning axle driven by an electric motor (see Experimental Procedures section for more details). **D, E:** A tendon-like construct before (D) and after (E) 4 days of stretching. During this time period, the construct doubled in length from 8 to 16 mm. All stretched constructs were stretched at the same rate by linking to a single rotating axle.

The chick third toe increases in length from 5.9 to 12.9 mm between embryonic days 10 and 14 (Fig. 1A) (as previously reported by Hamburger and Hamilton,^1992^). This approximate doubling of the third toe length over 4 days in ovo is accompanied by an increase in metatarsal tendon length. To mimic this increase in length in the tendon-like constructs, a stretching rig was constructed that performed slow continuous elongation of ten constructs simultaneously (Fig. 1B and C). Constructs were formed as described with T0 defined as the time at which the construct first becomes taught (Kapacee et al.,^2008^). Stretching was performed during 4 days to double the length of the construct (at 2 mm per day, from 8 to 16 mm, Fig. 1D and E). This time point at maximum stretch was designated T4.

### TEM Ultrastructural Characterisation of the Effect of Stretching

**Fig. 2:**
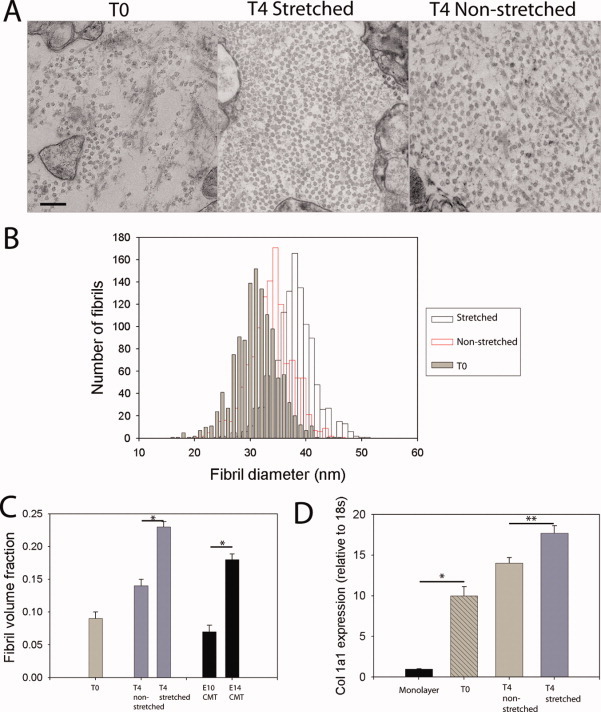
Effect of slow stretching on collagen matrix formation. **A:** TEM images at T0 and after four days with and without stretching. Collagen fibrils arranged in parallel can be seen in stretched and non-stretched samples. Scale bar = 250 nm. **B:** A fibril diameter histogram. Fibrils in the stretched construct show a shift toward increased fibril diameter (n=1,060 fibrils measured in each sample). **C:** Fibril volume fraction (FVF) increased between T0 and T4 in the non-stretched construct. However, the increase in FVF was greater after stretching. Values for day-10 and -14 ECMT are shown for comparison. **D:** Placing tendon cells in the fibrin gel (a 3D environment) resulted in significant up-regulation of *col1a1* expression at T0. Expression was further increased post-formation (between T0 and T4, *P* < 0.001). This change was more pronounced after stretching (*P* < 0.01). *P < 0.001, **P < 0.01. For TEM a minimum of two samples was examined for each condition; for qPCR three constructs were pooled and one experimental repeat performed. Error bars represent standard error of the mean.

TEM analysis showed the presence of collagen fibrils in the ECM in newly-formed constructs (T0) and after 4 days of culture both with and without stretching (Fig. 2A). Visual inspection of the TEM images suggested that the number of fibrils increased during 4 days in culture in both stretched and non-stretched constructs but that the fibrils appeared closer packed and better aligned with the axis of stretch in the stretched constructs. Quantitative analysis of a representative TEM survey showed that the mean fibril diameter increased from 32.1 ± 0.1 to 34.4 ± 0.1 nm in non-stretched constructs. However, the increase in fibril diameter was more pronounced in constructs that had been stretched (Fig. 2B). Here, the fibril diameters increased from 32.1 ± 0.1 nm to 38.4 ± 0.1 nm, and were significantly larger than in non-stretched samples (*P* < 0.001). The fibril diameters after stretching were similar to those seen in day-13 embryonic chick metatarsal tendon (32.1 ± 0.1 nm at day 10 increasing to 37.4 ± 0.2 nm at day 14).

The fibril volume fraction (FVF, the proportion of the extracellular space occupied by collagen fibrils) increased over 4 days in culture from 0.09 ± 0.02 to 0.14 ± 0.03 in non-stretched constructs (Fig. 2c). Stretching the tendon-like constructs for 4 days resulted in the FVF increasing by a further 79% (0.25 ± 0.05 in stretched constructs compared to 0.14 ± 0.03 in non-stretched constructs, *P* < 0.001). Correspondingly, the cell volume fraction (CVF, proportion of the transverse area occupied by cells) in constructs was 0.59 ± 0.01 at T0, and fell by 20% to 0.47 ± 0.01 at T4 in non-stretched constructs and by 7% to 0.55 ± 0.01 in stretched constructs. Thus, cells became more compact after stretching, resulting in a greater cell volume fraction.

Consistent with the increase in fibril diameter and fibril volume fraction, analysis of collagen gene expression demonstrated that stretching tendon constructs resulted in up-regulation of col1a1 gene expression (Fig. 2D).

### The Effect of Stretching on the Biomechanical Properties of Constructs

**Fig. 3:**
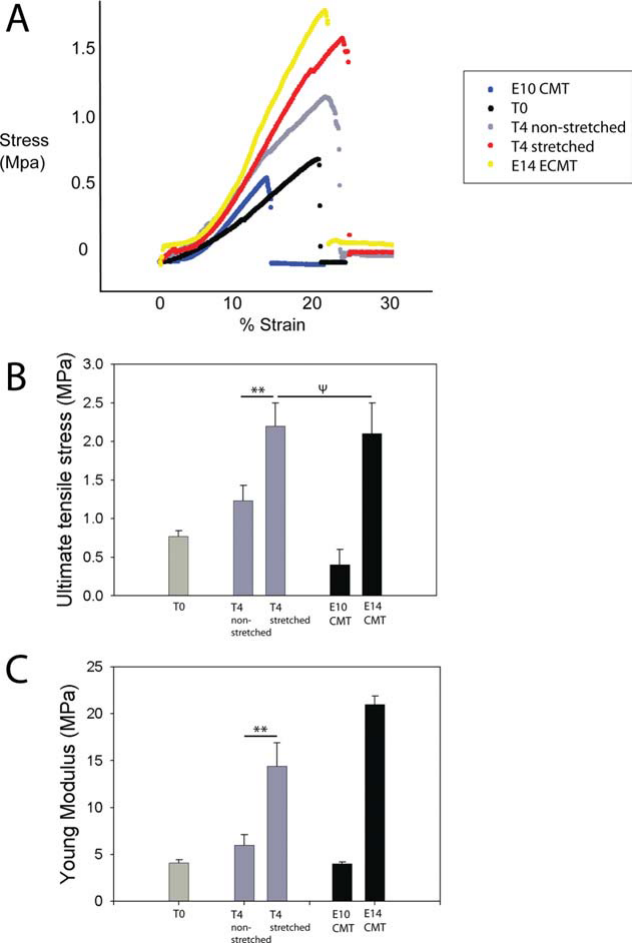
Mechanical properties of stretched constructs and embryonic chick metatarsal tendon. **A:** Typical stress-strain curves for chick tendon and stretched/non-stretched constructs. **B:** Ultimate tensile strength was increased in stretched constructs, which were not significantly different from day-14 embryonic chick metatarsal tendon. **C:** Young's modulus was increased in stretched constructs, but remained lower than day-14 embryonic chick metatarsal tendon. Values for day-10 and -14 tendons are shown for comparison. **P < 0.01; Ψ*P* > 0.1. For mechanical tests, eight samples were examined for each condition, and three separate experimental runs performed. Errors bars represent standard error of the mean.

Tensile failure tests were performed on tendon-constructs to determine the effects of stretching (during time in culture) on mechanical properties. Typical stress-strain curves for chick metatarsal tendons and tendon-like constructs are shown in Figure 3A. Ultimate tensile stress (UTS) increased 60% (from 0.77 ± 0.07 to 1.23 ± 0.18 MPa) without stretching, compared to an increase of 186% (2.20 ± 0.30 MPa) after stretching (*P* < 0.01, Fig. 3B). This was comparable with a UTS value of 2.12 ± 0.31 for day-14 chick metatarsal tendons (*P* > 0.1).

The Young's Modulus also showed a greater increase after stretching (202%), from 4.1 ± 0.3 MPa to 12.4 ± 2.5, compared with a 46% increase to 6.0 ± 1.1 in the non-stretched control (*P* < 0.01, Fig. 3C). The value after stretching was closer to the level of day-14 chick metatarsal tendons (20.5 ± 0.9).

### Modelling the Effect of Stretching on Fibril Length

The quantitative observations of increased fibril diameter and volume fraction can be interpreted in terms of a simple fibril growth model. It can be shown that the measured increase in diameters is insufficient to give the observed increase in fibril volume fraction and must also be accompanied by an increase in fibril length. The fibril volume fraction (FVF) depends on fibril diameter, fibril length, and number of fibrils per unit volume. FVF can be simply related to mean values of transverse area (A) and mean effective fibril length (L_eff_) by:


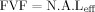
(1)

(*where N is the number of fibrils per unit volume*).

The change in FVF (ΔFVF), for constant N, is then related to the change in mean transverse area (ΔA) and mean effective length (ΔL_eff_) by:


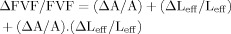
(2)

**Fig. 4:**
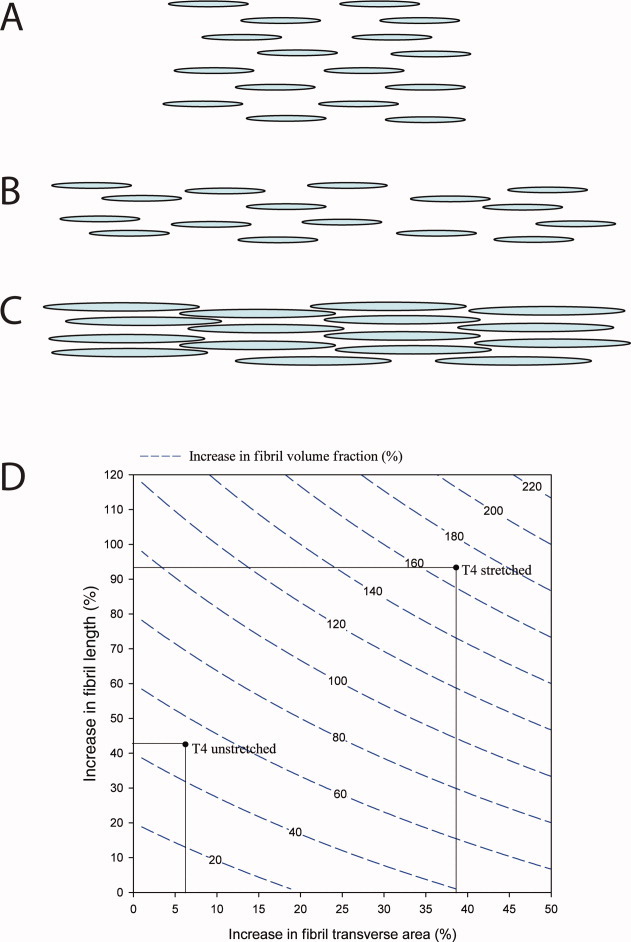
Model of the effect of stretching on fibril volume fraction. **A–C:** The effect of stretching the construct is shown schematically in 2D. A: An array of uniform collagen fibrils are shown. This array is extended in length in B while maintaining the fibril number, diameter, length, and volume fraction. C: The fibrils from the extended array are shown with increased diameter and length, both of which contribute to a higher fibril volume fraction. **D:** The predicted increase in fibril volume fraction is shown in the contour plot as a function of increases in fibril transverse area and fibril length. Two points (T4 non-stretched and stretched) are marked corresponding to the experimental fibril diameter and volume fraction data. There is a predicted increase in fibril length due to the stretching of the tendon construct as well as the directly observed increase in fibril transverse area.

This dependence is shown as a contour plot in Figure 4. The non-stretched construct shows a 7.2% increase in fibril transverse area and an increase of 55% in FVF. The predicted increase in fibril length is then 42%. The effect of stretching is to amplify these changes by 5-fold for fibril transverse area and 2-fold for fibril length. The fibril length increase could arise from an axial growth of existing fibrils, the formation and axial growth of new fibrils, or a combination of these two processes.

### Serial Section 3D Reconstruction

**Fig. 5:**
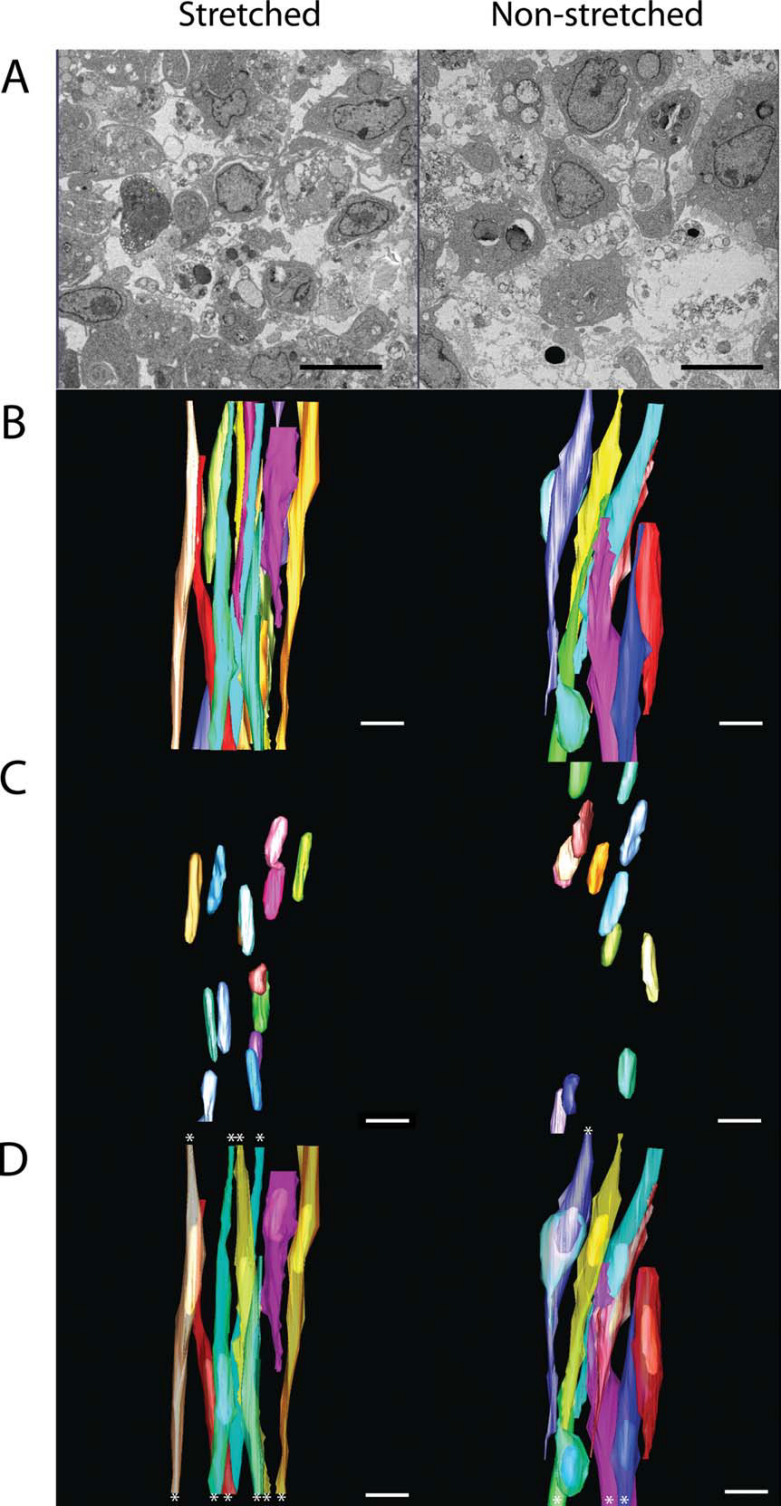
3D reconstructions of stretched and non-stretched constructs. **A:** A sample inverted back-scattered image. Scale bars = 5 μm. **B:** 3D reconstruction of cell plasma membranes demonstrates greater longitudinal cellular alignment in the stretched constructs. **C:** 3D reconstruction of cell nuclei. Measurements demonstrated elongation of nuclei in the stretched sample with no change in nuclear volume. These data are summarised in Table 1. **D:** Merged image of B and C. Cell plasma membranes (semi-transparent) and nuclei are shown. Stars mark cells that leave the 3D volume. Scale bar = 5 μm in A, 10 μm in B–D.

To generate a more complete understanding of the effect of stretching during growth in culture on the morphology of tendon-like tissue, 3D reconstructions were generated using serial block face imaging. A 40- × 40-μm region was imaged at 100 nm intervals over an 80-μm tissue depth (z range). Example images are shown in Figure 5A. A 3D reconstruction of cells and nuclei was generated for a stretched and non-stretched construct, using IMOD modelling software (Fig. 5B–D). The shape of the cells in stretched and non-stretched constructs was noticeably different. Only cells that were completely enclosed within the reconstructed volume were analysed. As a consequence, 15 cells in the stretched sample and 10 cells in the non-stretched sample were included in the analysis. For example, cells in stretched constructs were straighter and longer. Therefore, although it was easier to track individual cells in stretched constructs, and despite the relatively large z range investigated, some cells in the stretched constructs were not totally enclosed in the volume (Fig. 5B), indicating that some cells exceeded the z-depth of the reconstruction. In the stretched tendon-construct, 6-of-15 cells that were modelled went through both ends of the z volume and 9-of-15 went through one end. In contrast, cells in the non-stretched volume were not well aligned with the construct long axis and exited the sides of the volume. In the non-stretched sample, 0-of-10 cells went through both ends of the 100-μm z volume. By comparison, 5-of-10 cells were totally enclosed in the non-stretched control sample, with a mean length of 53.95 ± 12.78 μm. Furthermore, non-stretched cells had a greater cross-sectional area than stretched cells (Table 1). Taken together these data demonstrate that stretching results in changes in cellular morphology producing longer and thinner cells. Modelling of nuclei (Fig. 5C) revealed that that although nucleus volume remained constant, nucleus length was increased in stretched tendon-constructs (Table 1).

**Table 1:** Cell and Nuclear Shape Measurements Based on Reconstruction From Serial Section

	Stretched	Non-stretched	Change, %	Significance
**Cell cross-sectional area (μm**^**2**^)	**9.47**	**12.26**	29.5	*P* < 0.01
Standard error of the mean (SEM)	0.94	2.21		
**Nuclear cross-sectional area (μm**^**2**^)	**4.62**	**5.45**	−18.2	*P* < 0.01
SEM	0.27	0.24		
**Nuclear length (μm)**	**15.12**	**12.92**	17	*P* < 0.01
SEM	0.88	0.91		
**Nuclear volume (μm**^**3**^)	**90.17**	**89.43**	<1	*P* > 0.1
SEM	6.74	4.54		
**Cells totally within the volume**	0/15	5/10	—	—
**Cells leaving both ends of the volume**	6/15	0/10	—	—

## DISCUSSION

This study tested the hypothesis that the mechanical strain associated with growth is a key stimulatory factor for the observed changes in structural and mechanical properties of chick tendon during embryonic development. In vivo experiments in living embryos present challenges for the study of mechanical signal transduction, principally because it is difficult to isolate a single mechanical factor. Therefore, a cell culture system in which embryonic tendon cells synthesise a fibrous matrix with structural and mechanical properties similar to developing chick embryonic tendon was used. Subjecting this tissue to constant low-strain resulted in both structural and mechanical changes that matched, to a reasonable approximation, those seen over the same time period in the developing chick tendon. These changes included increased size and volume fraction of collagen fibrils (the principal load-bearing component in connective tissues) together with an increase in tissue strength and stiffness. These data provide evidence in support of the hypothesis that constant strain (presumably derived from bone elongation) is a key stimulus for the development of a mechanically functional tendon. However, additional factors such as growth factors originating from circulatory cells are also likely to make important contributions in vivo.

Early in embryonic development, collagen fibrils have narrow diameters (∼30 nm in embryonic tendon) but rapidly increase in size with the occurrence of fibrils with diameters in excess of 250 nm in P21 post-natal tendon (Humphries et al.,^2008^). It is thought that fibrils increase in diameter by molecular accretion (Holmes and Chapman,^1979^), or by inter-fibrillar fusion (Birk et al.,^1997^; Zhang et al.,^2005^). Here we report a gradual increase in collagen fibril diameter stimulated by low strain-rate and also an increase in fibril volume fraction (FVF). The scale of the increase in FVF indicates that there is an increase in fibril length, which accompanies the increase in diameter. At least part of this additional fibril length could be due to the nucleation of new fibrils. It is to be noted that an inter-fibrillar fusion process alone would not give an increase in the FVF but would require new fibril nucleation and a growth phase by molecular accretion.

The cell-shape is markedly changed by the slow stretching in that the cells elongate in proportion to the strain in the tendon-like construct while maintaining a near-constant volume. Concomitant with the change in cell shape, fibripositors form a primary mechanical interface between the cell cytoskeleton and the ECM (Canty et al.,^2006^). There is also a direct cell–cell mechanical linkage through cadherin-11-mediated junctions that stabilise an extended cell network (Richardson et al.,^2007^). Either or both of these mechanical interfaces (cell–matrix and cell–cell) could potentially transmit forces to elongate individual cells.

It is well established that mechanical factors can influence transcription and translation (Gieni and Hendzel,^2008^). Some studies show that forces can act directly on the nucleus, causing distortion of the nucleosome and gene expression changes (reviewed in Dahl et al.,^2008^; Buxboim et al.,^2010^). Nuclear membrane proteins (lamins) are linked to the cytoskeleton and lamin-deficient cells show impaired mechanotransduction (Lammerding et al.,^2004^). Manipulating nuclear shape by constraining cells on micro-patterned surfaces can alter expression of collagen type I collagen (col1a1) mRNA (Thomas et al.,^2002^). These studies show that gene expression can be modulated by mechanically-induced distortion of the nucleus, and it is postulated that nuclear distortion alters transcription factor affinity, nuclear traffic, chromatin organisation, and nuclear matrix organisation. It has also been shown that nuclei of stem cells are more deformable than differentiated cells (Pajerowski et al.,^2007^). Data obtained here from serial section EM demonstrate that embryonic tendon cell nuclei exposed to continuous low strain deform and stretch, become longer with reduced cross-sectional area, whilst maintaining a constant nuclear volume. These changes could indicate changes in tensile stress across the entire cell, which could lead to changes in gene expression, or could directly explain the observed up-regulation of gene expression and associated matrix development. Further studies using gene knock-down of nuclear membrane proteins and cytoskeleton-nucleus link proteins (such as emerin) will address if nuclear deformation has a direct role in development of the tendon ECM.

It is notable that after developmental day 14, the chick tendon increases dramatically in strength and stiffness. It is likely that other factors act in addition to slow stretching during development to enhance the mechanical properties of the ECM. Cyclic loading (principally provided by muscle contraction) results in diverse changes to cellular metabolic and biochemical processes (reviewed in Wang and Thampatty,^2008^) and it is likely that these processes are important during development (see Wozniak and Chen,^2009^). Similarly, hormonal and growth factor signalling, such as fibroblast growth factors (FGF) (Sheeba et al.,^2010^; Lejard et al.,^2011^) are important controllers of tendon development.

Unidirectional stretching of cells in collagen gels results in cell orientation parallel to the direction of stretch (Eastwood et al.,^1998^). This is consistent with findings in this study that cells in stretched constructs were more elongated and aligned with the direction of force. Skeletal muscle cells seeded on a silastic membrane put under linear strain of approximately 2% per day for 4–6 days aligned parallel to the direction of force and increased in length compared with muscle cells on non-stretched membrane (Collinsworth et al.,^2000^).

This study has investigated the effect of stretch on embryonic tendon-like tissue generated in vitro. Ultrastructural and mechanical analyses demonstrated that stretch results in enhanced development of a tendon-like construct. These data suggest that stretch resulting from limb elongation during rapid embryonic growth is an important mechanical stimulus for the development of embryonic tendon.

## EXPERIMENTAL PROCEDURES

### Cell Isolation and Tendon-Construct Formation

Tendon constructs were assembled as previously described (Kapacee et al.,^2008^, ^2010^). Briefly, day-13 ECMT cells were propagated (not exceeding passage 7) in monolayer in DMEM4 culture medium (Sigma, St. Louis, MO) supplemented with penicillin (100 U/mL, Sigma), streptomycin (100 μg/mL; Lonza, Basel, Switzerland), l-glutamine (2 mM; Lonza), and fetal calf serum (10%; Sigma) until sufficient numbers of cells were available to form constructs. Cells were removed from tissue culture flasks using trypsin–EDTA (Lonza). Each well of a six-well plate was lined with 2 mL of Sylgard (type 184 silicone elastomer, Dow Chemical, Midland, MI) and incubated at 55°C for 15 hr to set. Two 0.1-mm minutien pins (Austerlitz, Czech Republic) were each put through one 0.25-cm-length of suture (Ethicon, Cornelia, GA) and inserted with a 1-cm gap in the Sylgard. Plates were sterilised by immersion for 1 hr in 100% ethanol under UV light. Subsequently, 6.15 × 10^5^ cells were suspended in 400 μL of complete medium plus 83 μL of 20 mg/mL fibrinogen and 10 μL of 200 U/mL thrombin (both bovine; Sigma), deposited in each well and incubated at 37°C, 5% CO_2_. After a 5-min setting time, cell-matrix layers were scored with a fine pipette tip to prevent adhesion to the side of the well, then incubated with 5 mL culture medium (as above) supplemented with L-ascorbic acid 2-phosphate (200 μM; Lonza). The gel was scored every 2 days until, at approximately 7 days post-seeding, it had contracted to form a linear construct between the pinned sutures. The timepoint of “contraction” was defined as T0.

### Design and Construction of a Stretching Apparatus

An apparatus was constructed to perform simultaneous slow stretching of 10 tendon constructs. The stretching could be initiated after the tendon construct had formed and the stretching rate could be set and maintained over several days. Standard 6-well culture plates were modified as shown in Figure 1B and C. The two corner wells had mechanical link pins inserted to enable stretching and the four remaining wells were available for control tendon constructs. Five of these modified plates were mounted as a vertical stack on a rigid aluminium frame above a motor drive unit attached to the base plate as shown in Figure 1C. The sliding pins moved smoothly in PTFE collars and were connected by lengths of suture to the output shaft of a motor gearbox system as shown in Figure 1D. The motor supply voltage was set to achieve 3,400 rpm. The gearbox consisted of 12 reduction stages, each of ×4, with a final ratio of 1.68 ×10^7^: 1. The output shaft of 3-mm diameter rotated once in 4.7 days giving a take-up speed for the sutures of 8 mm in 4 days. This regimen was designed to reproduce the growth rate previously observed in the embryonic chick leg (Hamburger and Hamilton,^1992^). The motor unit was housed in a sealed Perspex enclosure with a container of drying agent to avoid high humidity/condensation problems of electrical instability and corrosion.

### Mechanical Testing

Constructs were removed from culture at contraction (T0) and at 4 days post-contraction (T0, T4 respectively). Eight tendon constructs were tested per time point as previously described (Kalson et al.,^2010^). Because it was not possible to take histological sections of constructs before mechanical testing (which would damage the tissue), construct diameters were measured from digital photographs. The diameter was then used to calculate transverse area according to the formula πr^2^. This assumed a circular transverse shape, as demonstrated previously from histological sections (Kapacee et al.,^2008^) and used in mechanical testing of tissue engineered ligament (Hairfield-Stein et al.,^2007^). An average of three diameter measurements was recorded for each construct. Tendon constructs were mounted on a supportive frame of grade 100 sandpaper using super glue. The mounting frame was clamped in an Instron 4301 mechanical testing machine fitted with a 100 N load cell (Instron Inc., High Wycombe, UK). After clamping, the side-pieces of the frame were cut to prevent pre-test stretching or damage of the tendon construct. Samples were excluded from analysis if they slipped during testing. The original contour length (LO) of tendon constructs was measured from a digital photograph of the mounted construct. A tare load of 10 mN was applied at the start of the tensile test to fully straighten the tendon construct. The length at failure was determined from the Instron test (giving change in length LΔ). Tendon constructs were tested to failure with a strain rate of 5 mm per minute (equivalent to approximately 1% strain per second). Tensile testing of embryonic chick metatarsal tendon, cut into 1.5-cm lengths, was also performed using this method, and a minimum of eight samples was tested per time point.

### Electron Microscopy

For ultrastructural analysis, a minimum of 2 constructs was examined for each time point. Constructs were immersed in primary fixative (100 mM sodium phosphate buffer [pH 7.0] containing 2% glutaraldehyde; Agar Scientific, Stansted, UK) for 30 min at room temperature, then cut up into three smaller pieces and placed in fresh primary fixative for 2 hr at 4°C. Samples were transferred to secondary fixative (50 mM sodium phosphate buffer [pH 6.2] containing 2% glutaraldehyde and 1% osmium tetroxide; Agar Scientific) for 40 min at 4°C before being thoroughly washed with distilled water and en bloc stained in 1% aqueous uranyl acetate for 16 hr at 4°C. Constructs were dehydrated in graded acetone (30, 50, 70, and 90%) followed by four changes of 100% acetone for 10 min each at room temperature. Samples were treated with propylene oxide for 10 min at room temperature, then infiltrated with a mixture of TAAB low-viscosity resin (medium hardness; Agar Scientific) and propylene oxide. Samples were put into 50% resin on a rotator overnight at room temperature. The sample was then incubated in 70%, 90% and three changes of 100% resin, each for 1 hr at room temperature. Samples were put in resin moulds for polymerization at 60°C for 24 hr.

### Sectioning and Staining

Ultra-thin sections were taken on a Reichert-Jung Ultracut (Leica Microsystems, UK) ultramicrotome using a diamond knife (Druker International, NL). Sections were stained with 2% uranyl acetate in 70% ethanol for 20 min, then washed in distilled water. Sections were counter-stained in 0.3% lead citrate in 0.1 M NaOH for 5 min and washed in distilled water.

### Micrograph Capture, Sampling Technique, and Ultrastructural Measurements

Sections were examined using an FEI Tecnai 12 Twin Transmission Electron Microscope (TEM). Images were captured using a 2 k × 2 k cooled CCD camera (F214A, Tietz Video and Image Processing Systems, Gauting, Germany). For each sample, a minimum of three different sections was reviewed. A thorough sampling of each section was performed. Three different magnifications were used: 2,100× for cell volume fraction (giving a large-area survey), 6,800× for fibril volume fraction, and 11,000× for fibril diameter. The sampling procedure generated 20 images of each section at 2,100×, 40 views per section at 6,800×, and 60 views per section at 11,000×. This sampling method enabled representative EM quantification of the collagenous matrix. Magnification calibration was performed for each magnification using a cross-grating replica grid (2,160 lines/mm, Agar Scientific, Stansted, UK). All measurements were made using ImageJ software (NIH freeware, http://rsb.info.nih.gov/nih-image). Cell volume fraction was calculated from the proportion of the transverse area occupied by cells (total transverse area/cell area). Fibril volume fraction was calculated from the proportion of transverse area occupied by collagenous fibrils (total transverse area/collagen fibril area).

### Serial Block Face Imaging

Samples were fixed in 2% glutaraldehyde (Agar Scientific) in 0.1 M cacodylate buffer (pH 7.2). To increase the back scattering potential of the sample, they were en-bloc stained in 1% osmium tetroxide, 1.5% potassium ferrocyanide in 0.1 M cacodylate buffer. This was followed by 1% tannic acid in 0.1 M cacodylate buffer. After washing, more osmium was added by staining in osmium tetroxide (1%) in 0.1 M cacodylate buffer. The final staining step involved soaking in 1% uranyl-acetate. The samples were dehydrated in ethanol and infiltrated in Araldite resin (CY212, Agar Scientific). Following embedding, the samples were sectioned using a Gatan 3view microtome within an FEI Quanta 250 scanning microscope. This technique allows a back-scattered electron image of the block face to be collected after each microtome cut (Denk and Horstmann,^2004^). For this particular experiment a 41- × 41-μm field of view was chosen and imaged using a 4,096 × 4,096 scan, which gave an approximate pixel size of 10 nm. The section thickness was set to 100 nm as the tendon cells are elongated and so less resolution is required in the Z (cutting) direction as opposed to the X and Y. Data sets were smoothed in IMOD to reduce imaging noise and models were created using the IMOD suite of tools (Kremer et al.,^1996^).

### Molecular Biology

The following primers were used: chicken Col1a1 forward primer, TGCT GTTGATAGCAGCGACT; reverse primer, GTGTCCTCGCAGATCACCTC; chicken ribosomal 18s forward primer, GTAA CCCGTTGAACCCCATT; reverse primer, CTACTACCGATTGGATGG. Total RNA was extracted from subconfluent (∼80%) cultures of fibroblasts using TRIzol reagent followed by DNase treatment. For RNA isolation and reverse transcriptase-PCR, tendon constructs were rinsed briefly in PBS. Three constructs were pooled for each condition examined, and two separate experimental runs performed. TRIzol reagent (Invitrogen, Carlsbad, CA) was added and the tissue rapidly frozen in liquid nitrogen prior to disruption using a Mikro-Dismembrator (Sartorius, Goettingen, Germany) (twice at 2,000 rpm for 90 sec). RNAs were extracted from the tissue following the manufacturer's instructions (Invitrogen) followed by DNase treatment. cDNA was transcribed from 2 μg of RNA with TaqMan reverse transcriptase (RT) polymerase (Applied Biosystems, Foster City, CA), using an oligo(dT)16 primer. RT-PCR analysis was performed on mRNAs by using 20-mer primers complementary to col1a1 and ribosomal 18s from the chick. Amplification of the correctly sized products was verified by electrophoresis on a 2% Tris–borate–EDTA gel. The identities of the product were confirmed by DNA sequencing. PCR products (5 ng) were sequenced using a BigDye Terminator v3.1 cycle sequencing kit (Applied Biosystems). Samples were placed in a thermal cycler under the following conditions: initial denaturation was performed at 96°C for 1 min, followed by 25 cycles of 96°C for 10 sec, 50°C for 5 sec, and 60°C for 4 min. Samples were precipitated with ethanol–sodium acetate prior to analysis. RNA was extracted and cDNA synthesized as described above. A total of 50 ng cDNA was loaded per qPCR well. Quantitative RT-PCR was performed using Chromo4 (BioRad, Hercules, CA) with SYBR Green (Eurogentec, Seraing, Belgium). Output was analysed using Opticon monitor 3 software (BioRad). Results were normalized to ribosomal 18s and four samples were used for each time point (Schmittgen and Livak,^2008^).

### Statistical Analysis

Statistical analysis was performed using SPSS version 14. Mechanical data (UTS, modulus, and failure strain) and ultrastructural data (fibril diameter, cell volume fraction, and fibril volume fraction) were examined using 1-way ANOVA. Quantitative PCR data was examined using the Wilcoxon two group test. Significance was set at the *P* < 0.05 level. Data are presented as mean ± standard error of the mean unless otherwise stated.

## ABBREVIATIONS

BSI: back-scattered imaging
CVF: cell volume fraction the fraction of the tissue occupied by cells
ECM: extracellular matrix
EM: electron microscopy
FVF: fibril volume fraction the fraction of the tissue occupied by collagen fibrils
SEM: scanning electron microscopy
